# Interactive role of diabetes mellitus and female sex in the risk of cholangiocarcinoma: A population-based nested case-control study

**DOI:** 10.18632/oncotarget.14254

**Published:** 2016-12-27

**Authors:** Yan-Jiun Huang, Alexander TH Wu, Hung-Yi Chiou, Ming-Tsang Chuang, Tzu-Ching Meng, Li-Nien Chien, Yun Yen

**Affiliations:** ^1^ Department of Surgery, Division of Colorectal Surgery, Taipei Medical University Hospital, Taipei, Taiwan; ^2^ The Ph.D. Program for Translational Medicine, Taipei Medical University & Academia Sinica, Taipei, Taiwan; ^3^ Graduate Institute of Cancer Biology and Drug Discovery, College of Medical Science and Technology, Taipei Medical University, Taipei, Taiwan; ^4^ School of Health Care Administration, College of Management, Taipei Medical University, Taipei, Taiwan; ^5^ School of Public Health, College of Public Health and Nutrition, Taipei Medical University, Taipei, Taiwan; ^6^ Institute of Biological Chemistry, Academia Sinica, Taipei, Taiwan

**Keywords:** cholangiocarcinoma, diabetes, population-based, intrahepatic, extrahepatic

## Abstract

Diabetes mellitus (DM) has been associated with an increased risk of extrahepatic cholangiocarcinoma (ECC) and intrahepatic cholangiocarcinoma (ICC). However, the role of DM in a population with a lower incidence of ECC remains unclear. We investigated the role of DM and other risk factors for ECC and ICC by conducting a population-based, nested, case–control study in Taiwan, a region with a lower incidence but a higher proportion of ICC. We identified patients who received a diagnosis of cholangiocarcinoma (CC) from the Taiwan Cancer Registry between 2003 and 2009. A total of 6,093 CC cases (ICC: 4,695; ECC: 1,396) and 60,906 matched controls were included. Compared with the controls, the patients with ICC and ECC were more likely to have DM, with an adjusted OR of 1.22 [95% confidence interval (CI): 1.07–1.39] and 1.48 (95% CI: 1.18–1.85), respectively. DM was associated with an increased risk of CC in the women and patients without a history of biliary tract diseases. Moreover, compared with the controls, DM was not associated with an increased risk of ECC in the patients who received cholecystectomy. These findings strongly support the positive association between DM and the increased risk of both ICC and ECC; however, this association was not observed in the patients who received cholecystectomy.

## INTRODUCTION

Cholangiocarcinoma (CC) is primarily a cancer of the epithelial cells (mostly adenocarcinoma) in the bile ducts arising anywhere along the intrahepatic or extrahepatic biliary tree [1, 2]. CC accounts for approximately 3% of all gastrointestinal cancers [3, 4]. The incidence rate of CC is high in Asia including Thailand and Korea [5]. In Western countries, extrahepatic CC (ECC) is the most common type, with 6%–8% of CCs being intrahepatic CC (ICC) and 27%–42% being ECC [1, 2]; this pattern in Western countries is different from that observed in East Asia [5].

Several epidemiological studies have reported that risk factors for ECC and ICC and the heterogeneity concept associated with ECC and ICC are different [5–8]. Numerous biliary tract diseases have been identified as risk factors for CC, including primary sclerosing cholangitis, liver flukes (*Opisthorchis viverrini* and *Clonorchis sinensis*) found in endemic regions such as Thailand, cholelithiasis, hepatolithiasis [3–5], and congenital biliary tract abnormalities such as choledochal cysts [3, 6, 7, 9]. Among these risk factors, pancreaticobiliary maljunction, cholelithiasis, and cholecystectomy have been associated with ECC [5]. By contrast, hepatitis B virus (HBV), hepatitis C virus (HCV), hepatolithiasis, and cirrhosis have been associated with ICC [5]. In addition, a study suggested that diabetes mellitus (DM) is associated with an increase in CC in Western countries [10]. However, its role in Asian countries remains controversial because of heterogeneity in the study design, selected study population, and study regions [11–17].

CC typically has a poor prognosis, with a 5-year survival rate of 11%–44% even after curative surgery [18]. Moreover, although CC is a rare malignancy in Western countries, it is more common in Asia. Because of an increasing prevalence of DM in Asia [19–20], understanding the role of DM in patients with ICC and ECC can be beneficial. In addition, identifying crucial risk factors for CC can help physicians in providing valuable preventive therapeutic strategies. Therefore, this study investigated the interactive role of DM in patients with ICC and ECC by performing a population-based, nested, case–control study.

## RESULTS

### Study sample

**Figure 1:**
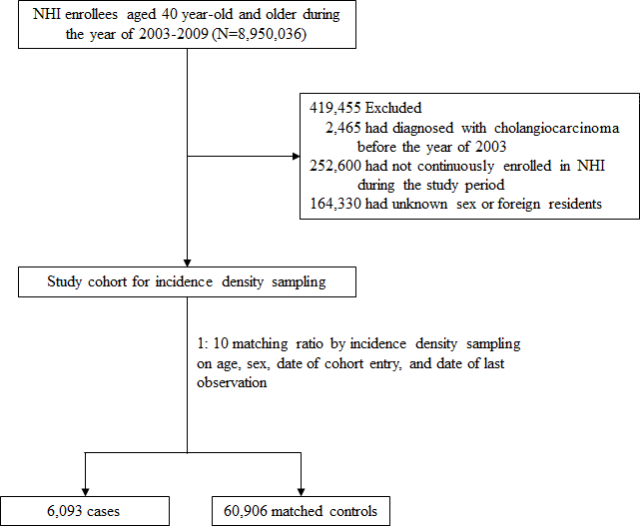
Flow Chart of the Selection Process of Cases and Matched Controls

The initial cohort consisted of over 8,000,000 NHI beneficiaries, and the final cohort consisted of 6,093 CC cases (ICC: 4,695; ECC: 1,396) and 60,906 age- and sex-matched controls (Figure 1).

### Basic characteristics

Table 1 lists the demographics, disease diagnoses, and medication use of the patients with ICC and ECC and their matched controls. The mean age of the patients with ICC and ECC was 67.8 (SD: 11.2) and 70.6 (SD: 11.4) years, respectively. The percentage of men was higher among the patients with ECC than among the patients with ICC (59.1% vs. 52.1%). Compared with the controls, both the patients with ICC and ECC were more likely to have DM, biliary tract diseases, cirrhosis, chronic pancreatitis, peptic ulcer, and CCI > 3 and receive *H. pylori* eradication therapy. Moreover, compared with the controls, the patients with ICC were more likely to have alcoholic liver disease, HBV, and HCV, whereas the patients with ECC were more likely to receive PPIs and NSAIDs.

**Table 1:** Characteristics of cholangiocarcinoma cases and matched controls

	ICC	Matched Controls		ECC	Matched Controls	
	N (%)	N (%)	*P*	N (%)	N (%)	*P*
Total	4,695(100)	46,942(100)		1,398(100)	13,964(100)	
Male	2,448(52.1)	24,480(52.1)		826(59.1)	8,252(59.1)	
Age(yr), mean(SD)	67.7(11.2)	67.8(11.3)		70.6(11.4)	70.7(11.4)	
Previous or coexisting medical condition						
Diabetes	978(20.8)	8,353(17.8)	**	309(22.1)	2,573(18.4)	**
Biliary tract diseases	619(13.2)	749(1.6)	**	195(13.9)	279(2.0)	**
Hemochromatosis	3(0.1)	22(0.0)		3(0.2)	11(0.1)	
Cirrhosis	191(4.1)	636(1.4)	**	33(2.4)	173(1.2)	**
Alcoholic liver disease	35(0.7)	155(0.3)	**	8(0.6)	47(0.3)	
Chronic non-alcoholic liver disease	25(0.5)	172(0.4)		7(0.5)	42(0.3)	
HBV	138(2.9)	513(1.1)	**	23(1.6)	164(1.2)	
HCV	112(2.4)	541(1.2)	**	24(1.7)	172(1.2)	
Chronic pancreatitis	9(0.2)	24(0.1)	**	4(0.3)	12(0.1)	*
Inflammatory bowel disease	22(0.5)	192(0.4)		8(0.6)	52(0.4)	
Peptic ulcer	955(20.3)	8,102(17.3)	**	323(23.1)	2,717(19.5)	**
GERD	114(2.4)	1,015(2.2)		31(2.2)	334(2.4)	
Cardiovascular disease	892(19.0)	8,947(19.1)		307(22.0)	3,092(22.1)	
Hyperlipidemia	611(13.0)	7,003(14.9)	**	209(14.9)	2,086(14.9)	
Charlson comorbidity index, mean(SD)						
0	2,079(44.3)	25,579(54.5)	**	583(41.7)	6,877(49.2)	**
1	1,097(23.4)	10,221(21.8)		352(25.2)	3,291(23.6)	
2	736(15.7)	5,370(11.4)		211(15.1)	1,770(12.7)	
≥3	783(16.7)	5,772(12.3)		252(18.0)	2,026(14.5)	
Medication						
H. pylori eradication therapy	620(13.2)	5,311(11.3)	**	287(20.5)	1,875(13.4)	**
PPIs	281(6.0)	2,635(5.6)		92(6.6)	792(5.7)	*
H_2_RA	121(2.6)	1,139(2.4)		44(3.1)	386(2.8)	
Aspirin	954(20.3)	9,977(21.3)		344(24.6)	3,447(24.7)	
NSAIDs	997(21.2)	9,826(20.9)		365(26.1)	3,284(23.5)	*
Statins	307(6.5)	3,491(7.4)	*	95(6.8)	1,103(7.9)	
Metformin	565(12.0)	4,893(10.4)	**	161(11.5)	1,472(10.5)	
Insulins	104(2.2)	820(1.7)	*	35(2.5)	278(2.0)	
Other antidiabetic drug	710(15.1)	6,036(12.9)	**	210(15.0)	1,904(13.6)	

Abbreviation: ICC, intrahepatic cholangiocarcinoma; ECC, extrahepatic cholangiocarcinoma; biliary tract diseases: choledochal cysts, cholangitis, cholelithiasis and cholecystitis; HBV, hepatitis B virus; HCV, hepatitis C virus; GERD, gastroesophageal reflux disease; PPIs, proton pump inhibitors; H_2_RA, histamine-2 receptor antagonists; NSAIDs, non-steroid anti-inflammatory drugs.

*P <0.05, **P <0.001, *P* value was based on the unadjusted regression analysis.

### Diabetes and risk factors for ICC and ECC

**Figure 2:**
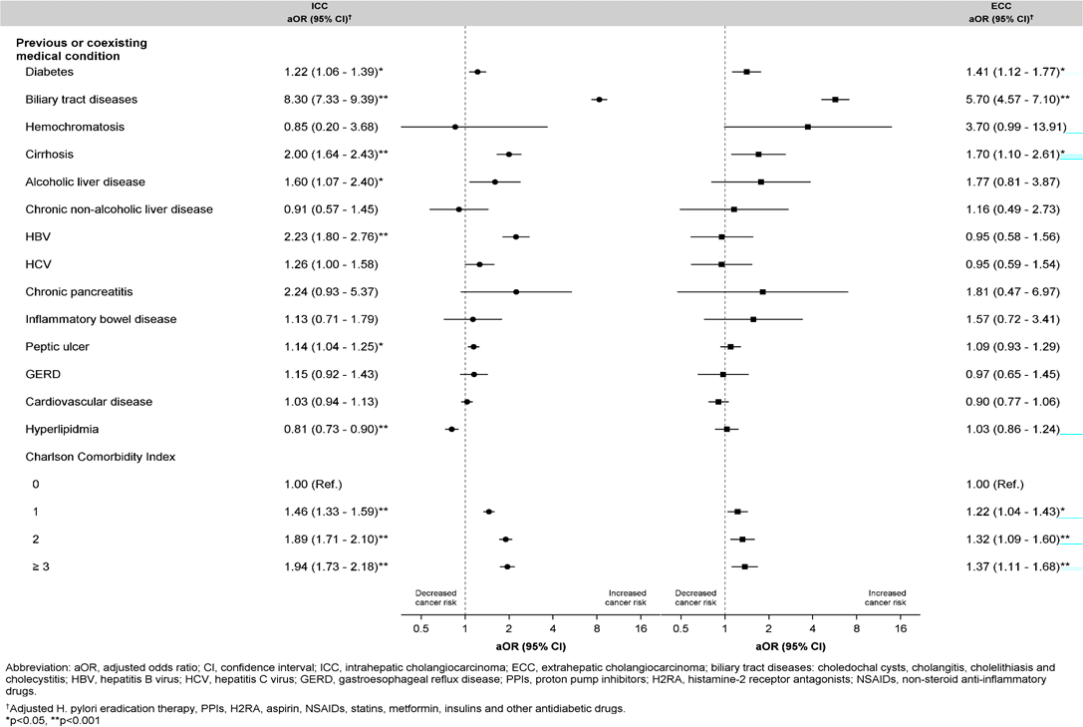
Adjusted Odd Ratios with 95% Confidence Interval for Previous or Coexisting Conditions Associated with ICC or ECC

Figure 2 presents risk factors for ICC and ECC. Compared with the controls, the patients with ICC and ECC were more likely to be exposed to DM, with an adjusted OR of 1.22 (95% CI: 1.07–1.39) and 1.48 (95% CI: 1.18–1.85), respectively. Biliary tract diseases and cirrhosis were also more likely to occur in the patients with ICC and ECC. However, compared with the matched controls, the higher odds of alcoholic liver disease, HBV, and peptic ulcer were observed only among the patients with ICC.

### Stratified analysis

**Figure 3:**
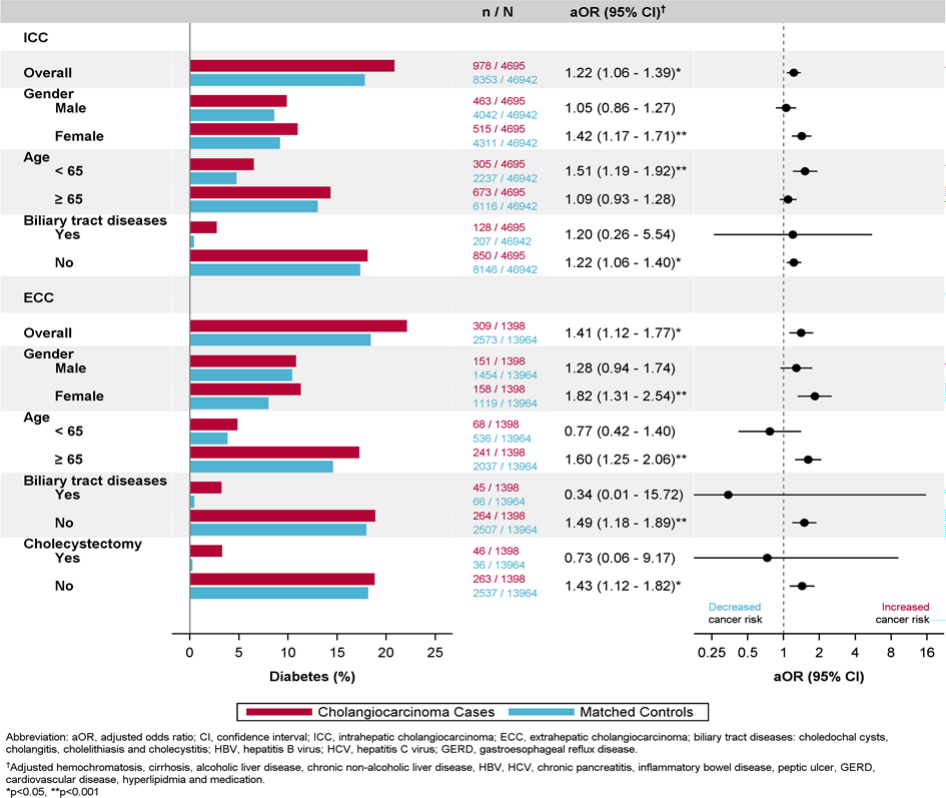
Subgroup-Specific Adjusted Odd Ratios With 95% Confidence Interval for the Risk of Diabetes Associated with Increased Risk of ICC or ECC

Figure 3 presents the adjusted odds of DM among different subgroups. The risk of DM was associated with ICC and ECC among the women, with an adjusted OR of 1.42 (95% CI: 1.17–1.71) and 1.82 (95% CI: 1.31–2.54), but not in the men. Compared with the controls, the risk of DM was more positively associated with ICC and ECC in the patients without biliary tract diseases. However, DM was not found to be associated with the risk of ECC in the patients who received cholecystectomy.

## DISCUSSION

The results of this study revealed that compared with the age- and sex-matched controls, both the patients with ICC and ECC were more likely to have DM after adjustment for all potential risk factors. In addition, in the subgroup analysis, the risk of DM was associated with ICC and ECC in the women but not in the men. Compared with the controls, the risk of DM was more positively associated with ICC and ECC in the patients without biliary tract diseases. However, DM was not found to be associated with the risk of ECC in the patients who received cholecystectomy.

The strength of this study is that it effectively explored differences in the association of risk factors for ICC and ECC with DM by using a nationwide, large sample-sized, and homogeneous population-based cohort. This approach prevents the selection and recall biases observed in previous case–control studies [11–15]. In addition, other strengths of this study are as follows. First, disease conditions, medications, and treatments were obtained from a single-payer insurance system with a comprehensive coverage. Second, multiple regression analyses were performed to adjust all potential confounding biases that can be observed in this cohort. The progress in the understanding of ICC has been limited by its rarity in Western countries. The ratio of ICC to ECC observed in our national cohort was quite different from that observed in Western countries. Although the ratio of the number of the patients with ICC (N = 4695) to the number of the patients with ECC (N = 1398) is high in our national cohort, unexplained rising incidence of ICC has been reported in studies conducted in Europe and the United States in recent years [7, 17, 18].

In general, the percentage of men was higher among the patients with ECC than among the patients with ICC (59.1% vs. 52.1%). Moreover, the mean age of the patients with ICC was higher than that of the patients with ECC.

Our study results revealed that compared with the controls, the patients with ECC were more likely to receive PPIs and NSAIDs. The association between long-term PPI use and carcinoma has remained questionable for years. A recent study reported an association of the long-term use of PPI with the risk of periampullary cancers [19]. Furthermore, studies have postulated that carcinoma is secondary to hypergastrinemia because of its use for over 15 years [19–20]. On the basis of these findings, two mechanisms underlying the association of ECC carcinogenesis with the use of PPI have been proposed. First, strong acid suppression causes an increase in serum gastrin levels. Prolonged and increased gastrin can stimulate an increase in intermediates that have trophic effects on the normal gastrointestinal mucosa, resulting in the stimulation of carcinogenesis. Second, Bernstein et al. suggested that bile acids act as carcinogens in human gastrointestinal cancers [21]. A long-term exposure to bile acids activates prosurvival stress-response pathways and affects several genes or proteins associated with chromosome maintenance and mitosis [22]. Therefore, a likely mechanism through which hydrophobic bile acids induce ECC is the bile acid induction of reactive oxygen species, reactive nitrogen species, and DNA damage in the cells of the gastrointestinal tract. If the stress induced by chronic inflammation is very high, it can also affect cellular defenses, resulting in cell death [23].

Biliary tract diseases including cholelithiasis and cholangitis are risk factors for both ICC and ECC. Patients with cholangitis or cholelithiasis have at least a 4-fold increase in the risk of CC [3, 6]. Gallstones induce biliary inflammation, and cholecystectomy is typically followed by the dilation of bile ducts, which might also cause inflammation and thereby possibly increase the risk of CC [24]. Although gallstone disease is common, only approximately 1% of patients with gallstones develop biliary tract cancer, implying that other factors may be involved in the pathogenesis of the cancer [25–26]. We particularly focused on IHC in this study because of the recent identification of a rising disease incidence in line with the rising number of DM cases in Asia. Previous studies on the association between DM and CC have yielded conflicting results [7, 11–13]. Our results demonstrated and confirmed that the risk of DM was positively correlated with ICC and ECC. Among our patients with ICC and ECC, the risk of diabetes was higher in the women with an adjusted OR of 1.42 (95% CI: 1.17–1.71) and 1.82 (95% CI: 1.31–2.54), respectively. Moreover, the patients with ICC were more likely to have diabetes, biliary tract diseases, and cirrhosis and receive *H. pylori* eradication therapy and metformin. The analysis of the SEER–Medicare databases indicated that DM was at least 2.5-fold more common in the patients with ICC than in the patients undergoing resection of hepatic colorectal metastasis, supporting the suggestion of the etiological role of DM [7].

Because DM is a public health issue worldwide, is most noticeable disease in developing countries, and has an increasing prevalence in the Asia Pacific, identifying the most susceptible population and protectong them from exposure to risks can be helpful [27]. In Taiwan, DM is a major health issue with an estimated 900,000 patients with DM among its 23 million local inhabitants. The annual incidence of DM has reached 0.5%–1% of Taiwan's total population, suggesting that there are at least 100,000 new cases every year. In addition, there has been a sharp rise in the prevalence of diabetes among Taiwanese citizens aged 65 years and older (from 8.7% in 1989 to 15.1% in 1999), as reported by the Taiwanese Department of Health [28]. Our data analysis demonstrated that compared with the controls, both the patients with ICC and ECC were more likely to have DM. Two Surveillance, Epidemiology and End Result (SEER) studies have also reported a positive association between diabetes and CC. In addition, Grainge et al. conducted a large, population-based, case–control study in the United Kingdom and demonstrated an association between diabetes and CC. After controlling for other risk factors in the stratified analysis of the risk of DM, we observed that DM itself was more pronounced in the nonbiliary tract disease group and also more significantly associated with bile duct cancer in the women than in the men. Men were reported to have a slightly higher rate of bile duct cancer than did the women, and our data support the previous finding and further demonstrate the pronounced association and influence of DM among the women in the context of ICC and ECC. The mechanisms through which the female sex plays a role in CC is believed to be related to the effect of hormone and its related receptors. Estrogen plays a crucial role in the pathogenesis of type 2 DM [29]. The estrogen level was higher in patients with CC [30]. Moreover, the estrogen level was increased in the serum of male patients with CC [31]. Two mechanisms through which estrogen or its receptor may play a direct or indirect role in the tumorigenesis of CC have been proposed. Estrogen acts on target tissues by binding to ESRs, ESR1 and ESR2, which have been detected in the tissues of the biliary tract [32–33]. Laboratory *in vivo* studies have indicated the presence of ESRs in the hepatobilary tree, including in bile duct epithelial cells, cholangiocytes, and CC cell lines [34–36], suggesting that estrogens play a role in CC carcinogenesis. Considering the potential hormonal role in biliary tract cancers, genetic variants in ESR1 and ESR2 genes have revealed an association between ESR polymorphism [ESR1 rs1801132 (P325P)] and bile duct cancer, which was more pronounced among patients with a low BMI or without biliary stones through their effect on estrogenic activity [36–37]. Recent studies have reported that FXR, a nuclear receptor for bile acids, plays a critical role in bile acid homeostasis as well as in glucose and lipid metabolism. The function of FXR is related to CC [38–41]. FXR consists of two primary domains: the DNA binding domain and the ligand binding domain [40–41]. Extranuclear signaling can be conveyed to the specific DNA site through these functional domains. A study on CC and its potential molecular mechanisms reported that the imbalance in the ratio of free to conjugated bile acids may play a crucial role in the tumorigenesis of CC [42].

*In vitro* experiments confirmed the clinical data because activator chenodeoxycholic acid (CDCA) stimulated the proliferation of ER-positive cells only in a steroid-free medium; the stimulation inhibited the siRNA-silencing of the FXR expression as well as the ER blockade by antiestrogens [43]. Furthermore, an *in vitro* study on co-immunoprecipitation reported that CDCA activated-FXR interacted with ER [43]. In our study, compared with the controls, the risk of diabetes was more positively associated with ICC and ECC in the patients without biliary tract diseases. However, DM was not associated with the risk of ECC in the patients who received cholecystectomy. The pronounced association and influence of DM within the female sex in the context of ICC and ECC are likely to be related to a crosstalk between FXR and ER in female patients with ICC and ECC [43]. Although no FXR expression has been reported in bile ducts, studies have reported that bile salts can regulate secretory activities in human gallbladder epithelial cells [44–45]. Obese patients with diabetes may have impaired emptying of the gallbladder even in the absence of gallstones. Bile acids regulate metabolism by binding to the nuclear receptor FXR. If gallbladders where FXR is located are removed surgically, DM cannot result in the formation of bile stasis or gallstones within the gallbladder. In turn, the biliary tree inflammation produced as a result of surgery is likely to cause a physiological response while attempting to repair it through cholangiocyte proliferation, which is a risk factor for CC.

Our results are partially in accordance with those of previous Asian studies that indicated a positive association between HBV and ICC [an adjusted OR of 2.23 (95% CI: 1.80–2.76)] [45]. HCV was also identified to be associated with ICC, which is in contrast to the finding of a study that reported a strong association in Western populations but not in East-Asian populations [46].

This study has several limitations that should be addressed. First, the data sets used in this study did not have information on risk behaviors, such as smoking and alcohol consumption, severity of comorbidities, and indication for medication; therefore, we could only adjust the presence of disease and medication use. In addition, we only considered the presence of diseases that occurred in the diagnostic claims; however, the time from when a disease starts to when it is identified or diagnosed usually lags. The latency time window of disease diagnoses might induce confounding of the association with cancer incidence by failure to account for disease duration. Therefore, the results should be interpreted cautiously. Finally, this study was conducted in the Pacific-Asian region; thus, our results might not be generalizable to other populations.

## MATERIALS AND METHODS

### Ethics statement

This study was approved by the Joint Institutional Review Board of Taipei Medical University (approval no. 201503054). Confidentiality was ensured by abiding to the data regulations of the Health and Welfare Data Science Center (HWDC), Ministry of Health and Welfare, Executive Yuan, Taiwan. To protect privacy, the HWDC provides patient-level data to investigators for research purposes only and encrypts individual identifiers before releasing the data to researchers. Informed consent of participants was exempted under the full review process of the Joint Institutional Review Board of Taipei Medical University.

### Data source

The Taiwan Cancer Registry (TCR), a population-based cancer registry, collects data of patients with cancer by using the standardized procedures of the registry's reporting system. TCR was started in 1979, and hospitals with 50 beds and more were required to report the basic information of patients who received a new diagnosis of cancer. Following the enactment of the Cancer Control Act in 2003, all reporting hospitals were mandated to submit cancer data to TCR. The database has a 2-year time lag between collection and publication because of quality-control audits according to the standard guidelines of the International Agency for Research on Cancer.

The Taiwan National Health Insurance Research Database (NHIRD), which is maintained by the National Health Insurance Administration (NHIA), covers almost all reimbursement claims received by beneficiaries under the regulation of the National Health Insurance (NHI) program. Since 1995, all the citizens of Taiwan are required by law to enroll in the NHI, and the coverage rate was up to 99% in 2012. The NHIRD contains patient-level claims data including inpatient, outpatient, and prescription drugs for disease diagnoses and treatments. To verify the accuracy of diagnoses and rationale for treatments, the NHIA routinely audits a proportion of the NHI claims. The two data sets can be linked using unique patient identification numbers.

### Case and matched control selection

From the TCR, we identified patients who received a diagnosis of CC between 2003 and 2009. Of these, we excluded patients who were aged less than 40 years, had received a diagnosis of other cancers in the observation period, were not continuously enrolled in the NHI since 2001, were foreign residents, or had unknown sex. Then, we classified patients into those with ICC and ECC on the basis of the International Classification of Disease of Oncology, Third Edition (ICD-O-3: C22.1 of ICC and C24.0 of ECC).

We selected up to 10 matched control patients without a history of cancer diagnosis during the study period from the study cohort. We used the incidence density sampling approach to match controls with each cancer case according to age (± 1 year), sex, date of cohort entry, and date of last observation (case occurrence, censoring, or end of the study) [21]. This method reduced not only the potential bias in observational studies but also the time-window bias by differentiating exposure opportunity time windows between patients [22]. The date of cancer diagnosis was considered as the index date. All control patients were assigned a pseudo index date (referred as the index date hereafter), which corresponded to the index date of their matched cases.

### Risk factors

DM was the major risk factor in this study. DM was defined if patients had two or more diagnostic claims of the International Classification of Disease, Ninth Revision, Clinical Modification (ICD-9-CM) code 250 in the NHIRD within 2 years prior to the index date. In addition, medication for treating patients with DM was also considered, including metformin, insulin, and other antidiabetic drugs based on their pharmacy claims. Patients were defined to have specific medication if they received a cumulative defined daily dose (cDDD) of more than 28 each year.

Other risk factors considered were biliary tract diseases (choledochal cysts, cholangitis, and cholelithiasis), cirrhosis, alcoholic liver disease, nonalcoholic liver disease, HCV, HBV, chronic pancreatitis, inflammatory bowel disease, peptic ulcer, gastroesophageal reflux disease, and cardiovascular diseases (atrial fibrillation, ischemic heart disease, congestive heart failure, and peripheral arterial disease) [5, 7]. In addition, we used Charlson's comorbidity index (CCI) that is classified into four grades, 0, 1, 2, and ≥3, as a measure of homogeneity and severity of clinical conditions between cancer cases and matched controls. Other medications considered included proton pump inhibitors (PPIs), histamine-2 receptor antagonists (H_2_RAs), aspirin, nonsteroidal antiinflammatory drugs (NSAIDs), and statins. The same approach used for defining patients with DM was adopted for defining patients having a specific disease condition or receiving drug medication.

We particularly focused on *Helicobacter pylori* infection; however, the diagnosis of *H. pylori* infection was underreported in the NHIRD. Thus, we considered whether patients received *H. pylori* eradication therapy to evaluate its association with CC. In addition, we identified patients who received cholecystectomy (surgical removal of the gallbladder). Cholecystectomy is a common treatment for symptomatic gallstones and other gallbladder conditions. A study reported an association of gallstones with the risk of ECC and ICC. However, whether cholecystectomy reduces the risk of ECC remains unclear.

### Statistical analysis

We investigated the association of the risk of DM with ECC and ICC by performing multivariate analyses with additional adjustments for potential confounders based on conditional logistic regression analyses. The odds ratios (ORs) and 95% confidence intervals (CIs) were determined. In this nested case–control study using incidence density sampling, the calculated estimated incidence ratio was roughly equal to the OR because of the low incidence of CC [23]. All analyses were performed using SAS/STAT 9.2 software (SAS Institute Inc., Cary NC) and STATA 12 software (Stata Corp LP, College Station, TX). A P value of <.05 was considered significant.
